# Dietary Patterns and Progression of Impaired Kidney Function in Japanese Adults: A Longitudinal Analysis for the Fukushima Health Management Survey, 2011–2015

**DOI:** 10.3390/nu13010168

**Published:** 2021-01-07

**Authors:** Enbo Ma, Tetsuya Ohira, Seiji Yasumura, Hironori Nakano, Eri Eguchi, Makoto Miyazaki, Mitsuaki Hosoya, Akira Sakai, Atsushi Takahashi, Hiromasa Ohira, Junichiro Kazama, Michio Shimabukuro, Hirooki Yabe, Masaharu Maeda, Hitoshi Ohto, Kenji Kamiya

**Affiliations:** 1 Health Promotion Centre, Fukushima Medical University, Fukushima 960-1295, Japan; teoohira@fmu.ac.jp (T.O.); m-miya@fmu.ac.jp (M.M.); mhosoya@fmu.ac.jp (M.H.); 2 Department of Epidemiology, Fukushima Medical University School of Medicine, Fukushima 960-1295, Japan; h-nakano@fmu.ac.jp (H.N.); e-eguchi@fmu.ac.jp (E.E.); 3 Radiation Medical Science Centre for Fukushima Health Management Survey, Fukushima Medical University, Fukushima 960-1295, Japan; yasumura@fmu.ac.jp (S.Y.); sakira@fmu.ac.jp (A.S.); junior@fmu.ac.jp (A.T.); jjkaz@fmu.ac.jp (J.K.); shima01@fmu.ac.jp (M.S.); hyabe@fmu.ac.jp (H.Y.); masagen@fmu.ac.jp (M.M.); hit-ohto@fmu.ac.jp (H.O.); kkamiya@fmu.ac.jp (K.K.); 4 Department of Public Health, Fukushima Medical University School of Medicine, Fukushima 960-1295, Japan; 5 Department of Paediatrician, Fukushima Medical University School of Medicine, Fukushima 960-1295, Japan; 6 Department of Radiation Life Sciences, Fukushima Medical University School of Medicine, Fukushima 960-1295, Japan; 7 Department of Gastroenterology, Fukushima Medical University School of Medicine, Fukushima 960-1295, Japan; h-ohira@fmu.ac.jp; 8 Department of Nephrology and Hypertension, Fukushima Medical University School of Medicine, Fukushima 960-1295, Japan; 9 Department of Diabetes, Endocrinology, and Metabolism, Fukushima Medical University School of Medicine, Fukushima 960-1295, Japan; 10 Department of Neuropsychiatry, Fukushima Medical University School of Medicine, Fukushima 960-1295, Japan; 11 Department of Disaster Psychiatry, Fukushima Medical University School of Medicine, Fukushima 960-1295, Japan; 12 Research Institute for Radiation Biology and Medicine, Hiroshima University, Hiroshima 734-8553, Japan

**Keywords:** chronic kidney disease, dietary pattern, eGFR, Fukushima Health Management Survey, proteinuria, trajectory analysis

## Abstract

To investigate associations between dietary patterns and the risk of impaired kidney function, we analyzed data from 14,732 participants (40–89 years) who completed the baseline diet questionnaire of The Fukushima Health Management Survey in 2011. The incidence of chronic kidney disease (CKD) (estimated glomerular filtration rate (eGFR) <60 mL/min/1.73 m^2^ or proteinuria (≥1+ by dipstick test)) and annual changes in eGFR were assessed from 2012 to 2015. Three major dietary patterns were identified. The adjusted cumulative incidence ratio of the highest vs. lowest tertile of a vegetable diet scores was 0.90 (95% confidence interval (CI): 0.82, 1.00) for eGFR < 60 mL/min/1.73 m^2^, 0.68 (95% CI: 0.52, 0.90) for proteinuria, and 0.88 (95% CI: 0.80, 0.97) for CKD (*P* for trend = 0.031, 0.007, and 0.005, respectively). The incident risk of CKD in the highest tertile of juice diet scores was 18% higher than the lowest tertile. The odds ratio of the highest vs. lowest tertile of vegetable diet scores was 0.85 (95% CI: 0.75, 0.98) in the rapidly decreasing eGFR group (*P* for trend = 0.009). We did not observe significant associations for the meat dietary pattern. A Japanese vegetable diet could reduce the risk of developing impaired kidney function and CKD.

## 1. Introduction

In 2017, the global prevalence of chronic kidney disease (CKD) was 9.1% (697.5 million cases), and deaths from CKD were 1.2 million, which was an increase of 41.5% from 1990 [1]. Approximately 7.6% (1.4 million) of deaths from cardiovascular disease (CVD) could be attributed to impaired kidney function. Patients with CKD have similar mortality risks and medical costs compared to those with CVD [2,3]. Between 1990 and 2017, CVD mortality declined by 30.4%; however, a decline in CKD mortality was not observed [1]. 

CKD is rarely diagnosed in the early stages, and the health-related quality of life in patients decreases with CKD progression [4]. Patients with CKD, especially those with dialysis treatment, are at increased risk of osteoporosis and suffering from bone pain and fractures [5]. Most patients with CKD 3 to 5 stages show an increased serum parathyroid hormone level [6]. In CKD-mineral and bone disorders, circulating factors released from vessels may affect bone metabolism, while the bone disease may increase vascular calcification, highlighting the mortality risk [6,7]. 

In kidney function impairment, the persistent low-grade inflammation has a prominent feature, and gut microbiota dysbiosis is a source of microinflammation [8]. Studies indicated that the most harmful uremic toxins produced by the gut microbiota are protein-bound and are recalcitrant to removal by dialysis [9]; the function of uremic toxins such as indoxyl sulfate may play roles in CVD development through altered monocytes activation, intensified inflammatory process, and augmented oxidative stress [10,11]. In addition, CKD and Type 2 diabetes have similar negative effects on both intestinal microbiota and function [12]. A recent animal study reported an increase in concentrations of uremic toxins in serum associated with a stronger impairment in cognition and higher permeability of the blood–brain barrier [13].

For the prevention of CKD, dietary control is among the modifiable factors receiving increasing attention. Dietary balance between acid-producing foods and alkaline-producing foods is important [14]. Given than some nutrients are highly correlated or interactive, studies on dietary patterns focusing on multiple food groups provide results resembling actual eating behaviors more than those studies based on a single dietary product or nutrient [15,16,17].

Dietary patterns such as the Mediterranean diet (higher intake of fruits, vegetables, legumes, cereals, and fish) [18,19,20], the Dietary Approaches to Stop Hypertension (DASH)-style diet (higher intake of fruits, vegetables, and whole grains) [21], and other diets [22] have been reported to be associated with a lower risk of estimated glomerular filtration rate (eGFR) in healthy adults in Western countries. Only a few studies on dietary patterns associated with loss of kidney function have been performed in Asian populations [23,24,25]. 

In Japan, CKD mortality is the eighth leading cause of death [1]. The Great East Japan Earthquake in March 2011 affected the health status of the residents in Fukushima, with increased cardiometabolic (e.g., overweight/obesity, hypertension, diabetes mellitus, and dyslipidemia) and possibly CKD risks [26,27]. In a previous study, we elucidated that a vegetable diet was inversely associated with cardiometabolic risks [28]. However, the association between dietary patterns and CKD risk has not been intensively investigated in general populations in Japan. 

In this study, we investigated the associations between dietary patterns identified by the Fukushima Health Management Survey (FHMS) in 2011 and the risks of developing impaired kidney function and CKD in the follow-up years until 2015 in Fukushima, Japan. 

## 2. Materials and Methods

### 2.1. Study Participants

The FHMS was initiated in 2011 after the Great East Japan Earthquake. The target population comprised 210,189 residents living in evacuation zones along with the radiation disclosure areas [29]. The FHMS included a self-administered questionnaire on socio-economic and demographic information, medical history and clinical treatment, lifestyle behaviors, and a food frequency questionnaire (FFQ) (*n* = 88,613). We used data from individuals aged between 40 and 90 years who had completed both the FFQ and a comprehensive health checkup in the 2011 fiscal year (*n* = 28,602). Partial participants who completed the health checkup in 2014 and 2015 were also utilized in the previous study [28]. 

### 2.2. Dietary Intake Assessment 

We used a short-form FFQ with 19 food items to determine the participant’s food intake during the 6 months preceding the survey date. The FFQ was a validated and modified version of the Hiroshima and Nagasaki Life Span Study [30]. In the validation study of the original FFQ, the frequency of food intake as measured by the FFQ was moderately correlated with food intake as measured by the 24-h recall records; for example, the Spearmen correlation coefficient of fruit, milk, miso soup, beef/pork, rice, and bread was between 0.14 and 0.34 [30]. The food items included non-juice fruits, non-juice vegetables (green vegetables, red and orange vegetables, and light-colored vegetables), fruit juice, vegetable juice, meat (chicken, beef/pork, and ham/sausages), soybean product (fermented soybean, soy milk, miso soup, tofu, and boiled beans), fish (raw and cooked), dairy (milk, yogurt, and lactobacillus drinks), rice, and bread. We asked the participants how often they consumed individual food items, with 6 response choices for frequency: none, <1 time/week, 1–2 times/week, 3–4 times/week, 5–6 times/week, or every day. 

### 2.3. End-Point Determination

We retrieved data on participants with at least 1 comprehensive health checkup conducted between the 2012 and 2015 fiscal years. Urinalysis by the dipstick method was conducted for a single spot urine specimen. The results of proteinuria testing were recorded based on the guidelines of the Japanese Committee for Clinical Laboratory Standards (http://jccls.org/) [27]. Serum creatinine was assayed using the enzymatic method. We calculated the eGFR using the Modification of Diet in Renal Disease formula recommended by the Japanese Society of Nephrology [31]: eGFR (mL/min/1.73 m^2^) = 194 × Cr^−1.094^ × age^−0.287^ × 0.739 (if female). We defined CKD as an impaired kidney function with an eGFR < 60 mL/min/1.73 m^2^ and/or proteinuria (≥1+ by dipstick test) [32].

### 2.4. Other Variables

Concerning covariates at baseline in 2011, we classified education status as “less or more than vocational university”; smoking history as “never, former, or current”; and alcohol consumption as “never, occasional, or regular”. We grouped physical activity into “none, 1 time/week, 2–4 times/week, or every day” and resident status at post-earthquake into “living in a shelter or temporary house, an apartment or rental house, or at relatives” or own house”. We assessed the mental health status of the participants with the Japanese version of the Kessler Psychological Distress Scale (K6). Scores ranged from 0 to 24, and we defined nonspecific distress as corresponding to a K6 score of ≥13 [33]. 

Blood pressure was measured with a standard sphygmomanometer or an automated device by medical staff at local institutes. We defined the participants’ cardiometabolic factors as follows: overweight as body mass index (BMI) ≥25 kg/m^2^; hypertension as systolic blood pressure (SBP) ≥140 mm Hg, diastolic blood pressure as ≥90 mm Hg, or the use of antihypertensive medication; and diabetes mellitus as fasting plasma glucose ≥126 mg/dL, hemoglobin A1c (HbA1c) ≥6.5% or the use of insulin or other medications. Participants who met the following criteria were diagnosed with dyslipidemia: hypo-high-density lipoprotein (HDL) cholesterolemia (as HDL-C < 40 mg/dL), hyper-low-density lipoprotein (LDL) cholesterolemia (as LDL-C ≥ 140 mg/dL), or hypertriglyceridemia (as high triglycerides ≥ 150 mg/dL). 

### 2.5. Statistical Analysis

**Figure 1:**
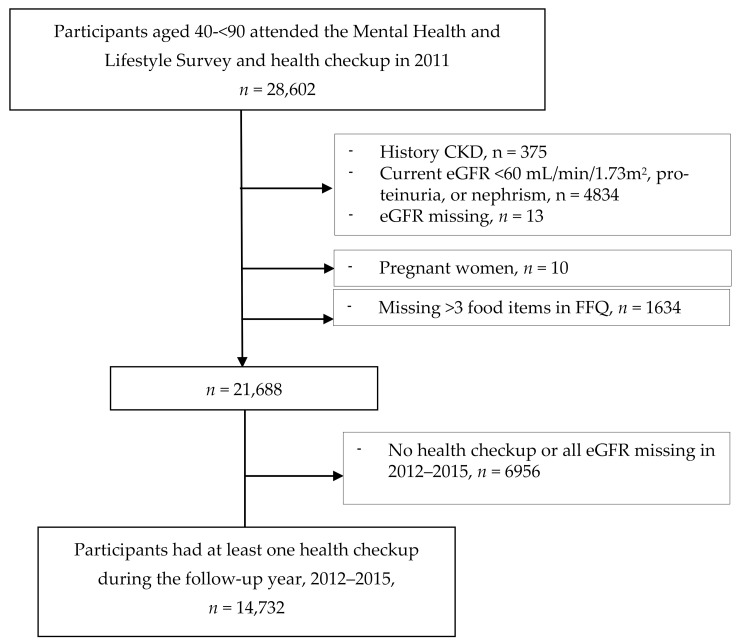
Flow diagram of study participants, Fukushima Health Management Survey, 2011–2015. CKD, chronic kidney disease; eGFR, estimated glomerular filtration rate; FFQ, food frequency questionnaire.

In the FFQs, we excluded pregnant women, participants with more than 3 missing FFQ answers [34], those with underlying kidney disease or abnormal kidney function, and those with missing estimated glomerular filtration rate (eGFR). In all, 14,732 individuals underwent the baseline survey and a health checkup in 2011 and at least 1 health checkup in the follow-up years (Figure 1).

For the remaining participants, those who did not answer some dietary questions (12.4% missed 1 and 4.2% missed 2 in the FFQ), we replaced the missing values with the sex-specific median value of that food item frequency [34]. For the intake frequency of each food item, we used the daily midpoint for the frequency category; e.g., we assessed “3–4 times/week” as 0.5 times/day [34]. 

We derived dietary patterns from food items using the principal component method of factor analysis. To achieve a simpler structure with greater interpretability, a varimax rotation was performed. We selected factor numbers mainly according to eigenvalues > 1.5, scree plots, and factor interpretability concerning food items, with absolute factor loadings ≥ 0.3 to account for each component [35]. Among 5 factors that satisfied the criteria, a 3-factor solution appeared to describe the most meaningfully distinctive dietary patterns of the study population. We labeled derived dietary patterns as “vegetables”, “juice”, and “meat” based on food items with high factor loadings on each pattern. Eigenvalues of the vegetable, juice, and meat patterns were 4.02, 1.72, and 1.55, respectively. The cumulative variance explained was 38.4%. Cronbach’s alpha coefficient for each dietary pattern indicated higher internal reliability of these measures: 0.79 for vegetables, 0.80 for juice, and 0.81 for meat. Given we derived almost the same factors from the factor analysis by sex, we only reported dietary patterns for total participants. We assigned each participant a pattern-specific score, which we calculated as the sum of the products of the factor loading coefficients and the standardized intake of food items. The factor scores reflect how closely a participant’s diet resembles each identified pattern, with higher scores representing closer resemblance [21]. We categorized the dietary pattern scores into tertiles for further analysis.

We calculated the annual eGFR change rate by the differences between baseline and final measurements and divided them by the follow-up time [36]. In the trajectory analysis for eGFR changes over time, the participants were classified into 3 groups: the group in which the eGFR level decreased most was defined as the early decliner group [37]. 

To compare baseline characteristics across groups, we used the chi-squared test for categorical data and an analysis of variance Kruskal–Wallis test (for median) and F test (for means) for continuous variables. We used the Poisson regression model to estimate the cumulative incidence ratio (CIR) between means of dietary pattern scores (with the first tertile as the reference) and the risk of impaired kidney function in the follow-up years. We applied the multiple linear regression model to measure the annual eGFR decline rate associated with dietary patterns. Finally, we performed a polytomous logistic regression analysis to examine the association between dietary pattern scores and groups of eGFR decliners, using the moderate lowering eGFR group as a reference [36]. We input the tertiles of each dietary pattern score simultaneously in the model, and the associations with outcomes were adjusted for age (continuous) and sex (Model 1); further adjustments were made for education, smoking history, alcohol consumption, physical activity, resident status, distress scale, BMI, diabetes, hypertension, and dyslipidemia by categories as mentioned above (Model 2). We selected these variables for adjustment based on previous publications for FHMS and clinical relevance [26,27,28,34,38,39]. Tests for trend were performed using median pattern scores in the tertile categories as continuous variables. Given that age is a strong factor in kidney function progression, we also added an age square for adjustment in modeling when both the age and age square were statistically significant. We performed a sensitivity analysis for participants who attended all the follow-up health checkups and for participants without cardiometabolic risk factors. 

We analyzed all the data using SAS statistical software version 9.4 for Windows (SAS Institute, Cary, NC, USA). All *p*-values reported were 2-sided, and *p* < 0.05 was considered statistically significant.

## 3. Results

Table 1 shows three independent dietary patterns with a minor overlapping of food group loadings. The vegetable diet pattern includes vegetables (white, green, red, and yellow vegetables), fish, fruits, bean products (tofu, fermented beans, boiled beans, and miso soup), and rice. The juice dietary pattern includes vegetable juice, fruit juice, yogurt, soymilk, fruits, milk, boiled beans, bread, and red/yellow vegetables. The meat dietary pattern includes chicken, beef/pork, and ham/sausage, and bread. 

Table 2 shows the social and demographic characteristics and health checkups of participants at the baseline. Comparing with men, women had higher education, less current smokers, less current alcohol drinkers, and higher dietary pattern scores but less frequent physical activity and more depression. The health checkup conditions were better in women than men, except for the LDL level. The elderly were more likely to follow vegetable and juice diets, not a meat diet. Those with higher consumption of the juice and meat diets were likely to have higher education and to exercise frequently. Current smokers and alcohol drinkers were less likely to follow vegetable and juice diets, with an inverse tendency of following a meat diet. Residents in temporary houses or shelters were likely to have lower consumption of vegetables (Table S1).

The SBP level, hypertension proportion, and fasting blood glucose level were higher in participants with a higher intake of vegetables but lower in participants with a higher intake of meat. Furthermore, participants with hyper-LDL-C showed an inverse tendency in consumption of a vegetable and juice diet. Participants with higher triglyceride levels declined along the ascendant tertiles of all the dietary patterns. 

The mean (standard deviation) eGFR at baseline was 75.7 (11.0) mL/min/1.73 m^2^; then, it gradually declined to 70.8 mL/min/1.73 m^2^ in 2015 (2.7 ± 1.2 follow-up years) (Table 3). Participants with eGFR 60–90 mL/min/1.73 m^2^ decreased from 89.1% in 2011 to 78.7% in 2015; in contrast, participants with eGFR < 60 mL/min/1.73 m^2^ increased to 14.9% in 2015. Compared with impaired eGFR level, proteinuria occurred much less frequently, showing a slight increase over time. The mean annual changes in eGFR rate during the follow-up years declined.

Table 4 shows the associations between dietary pattern scores and the risk of impaired kidney function. In Model 2, the highest vs. lowest tertile of a vegetable diet had a 10–32% reduced risk of development of eGFR < 60 mL/min/1.73 m^2^ and/or proteinuria with significant decreasing trends. The highest vs. lowest tertile of a juice diet increased the risk of developing impaired kidney function by 18–19% with significant increasing trends. No significant associations between a meat diet and impaired kidney function or CKD were observed.

**Figure 2:**
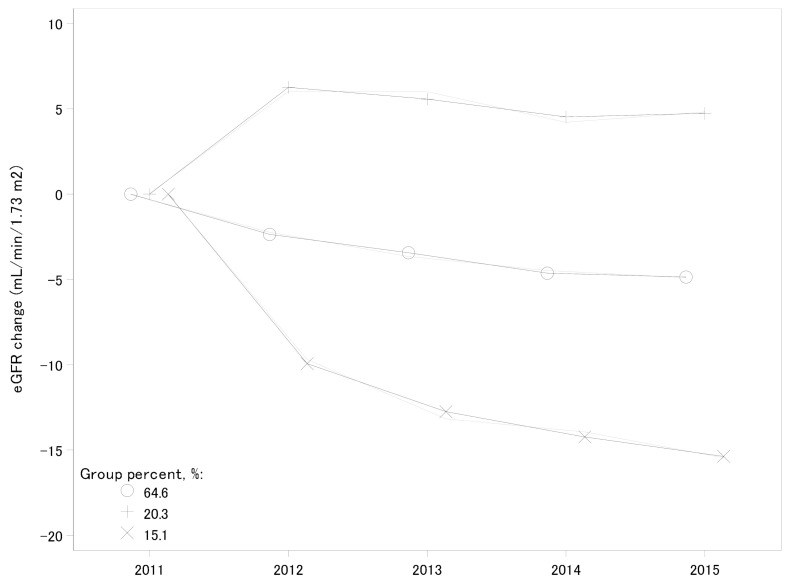
Trajectory groups of eGFR change over time, Fukushima Health Management Survey, 2011–2015. Solid lines were observed trajectory groups and short-dash lines indicate the model estimates. eGFR, estimated glomerular filtration rate.

Changes of eGFR in participants in the follow-up years were divided into three groups by trajectory analysis (Figure 2). We classified eGFR variance as an increasing group (20.3%), a moderate decline group (64.6%), and a rapid decline group (15.1%). The trajectory of the increasing group and the rapid decline group were approximately quadratic curves, whereas that of the moderate decline group was an almost straight line. Among the rapid decline group, most participants had higher risk categories, such as hypertension (14.7% vs. 12.6% nonhypertension), diabetes (17.9% vs. 13.1% nondiabetes), and living at temporary house or shelter (15.4% vs. 12.5% living in other places) (Table S2). For the yearly declines in eGFR, the highest vs. lowest tertile of a vegetable diet could prevent 15% of participants from rapid decline compared with the moderate decline eGFR group (Table 5).

In the sensitivity analysis for participants with all available kidney function data in the follow-up years (*n* = 4440), we observed similar significant associations between each dietary pattern and impaired kidney function or CKD. In the multivariable adjustment models, the CIR of CKD in the highest vs. lowest tertile was 0.83 (95% CI: 0.71, 0.97) (*P* for trend = 0.006) for the vegetable diet; 1.16 (95% CI: 1.00, 1.34) (*P* for trend = 0.038) for the juice diet; and 0.95 (95% CI: 0.83, 1.10) (*P* for trend = 0.507) for the meat diet. 

We repeated the regression analysis for participants with normal BMI, without hypertension, diabetes, or hyperlipidaemia; we observed similar significant associations between the vegetable and the juice dietary patterns and impaired kidney function (Tables S3–S5). However, the significant associations (CIRs) in participants with a normal BMI or without hypertension were attenuated to nonsignificance for the vegetable pattern. Other results in participants with hypertension had similar significant associations (CIRs) with CKD as in the main analysis. Participants with overweight and dyslipidemia showed similar significant associations (CIRs) with CKD for the juice pattern as in the main analysis, whereas the significant associations in participants with diabetes were attenuated to nonsignificance (data not shown).

## 4. Discussion

In this large population-based prospective study, we observed significant inverse associations between the vegetable dietary pattern and risks of impaired kidney function or CKD, and significant positive associations between the juice dietary pattern and eGFR < 60 mL/min/1.73 m^2^ or CKD. We also elucidated that the intake of a vegetable diet was inversely associated with annual eGFR changes, particularly in the rapid decline group.

The three dietary patterns identified in this study were similar to those in other Japanese studies [40,41,42,43]; particularly, the vegetable pattern had the most similar characteristics of high intake in the reproducible healthy/prudent patterns, i.e., the combination of vegetables, fish, fruits, bean products, and rice, in the typical Japanese diet [44,45,46]. In the Nurses’ Health Study in the USA, the DASH-style diet was inversely associated with eGFR decline ≥30% [21]. In a Swedish population, the medium and high adherents to the Mediterranean Diet were 23% and 42% less likely, respectively, to have CKD compared with the low adherents [19]. A recent meta-analysis has reported that the adherence to a healthy dietary pattern (rich in whole grains, vegetables, fruit, legumes, nuts, and fish, and a lower intake of red and processed meats, sodium, and sugar-sweetened beverages) was associated with lower odds of incident CKD and albuminuria [47]. Our study results support these findings and add evidence to these favorable associations in a general Japanese population. 

The anti-inflammatory mechanism underlying the beneficial effects of these diets could help reduce the incidence of chronic diseases, improving cardiometabolic profiles [11,18,48] and reducing metabolic acidosis [12,49]. A diet high in cereal fiber was protective against the development of moderate CKD among older adults [50]. High polyunsaturated fatty acid intake has been considered to be renal-protective [50,51]. Japanese and Mediterranean diets similarly feature seafood, vegetables, and fruits, as well as the soybean and soy products that are popular among the Japanese [52,53]. The Japanese dietary pattern is reportedly associated with the intake of antioxidant vitamins, minerals, dietary fiber, and omega-3 fatty acids [46,53]. Dietary intakes of omega-3 marine polyunsaturated fatty acids [54], soy, and isoflavones [55] were inversely associated with the incident risk of ischemic heart disease. Soybeans, a major source of plant protein, are associated with lower CVD mortality [56]. Moreover, the low consumption of sweeteners and the high consumption of green tea could be related to obesity status [57]. Therefore, the adoption of a healthy dietary lifestyle, lowering inflammatory markers, and/or dietary acid loading as mediators of cardiometabolic factors, e.g., preventing or stopping the vicious cycle between the gut microbiota and the cardiovascular/renal systems, might better preserve renal function, thus decreasing the morbidity and mortality of CVD and CKD [10,11,19,21,47,58]. 

We observed a significant positive association between the juice dietary pattern and eGFR < 60 mL/min/1.73 m^2^ and CKD. These results were similar to an Iranian study in which the dietary pattern of high fat and sugar was associated with a significant 46% increased risk of incident CKD [24]. Our results were also similar to the Nurses’ Health Study, in which the Western diet showed a positive association with microalbuminuria [21]. This result might be attributable to the higher intake of dairy products and sugar-based juices, such as the high-dairy [43] and bread [59] dietary patterns. The juice pattern in this study can be considered the reverse of the traditional Japanese staple food pattern [59]. The significant relationships between dietary patterns and inflammatory markers such as C-reactive protein could explain the direct association of the juice pattern with proteinuria [14,21]. 

High saturated fatty acid intake can deteriorate kidney function by affecting plasma creatinine [21]. The Western diet is rich in advanced glycation end products, formed when food is processed at increased temperatures, which is independently associated with the GFR [60]. Of note, the low saturated fat (meat) and high omega-3 polyunsaturated fat (fish) in the Japanese diet contribute to the low prevalence of hypercholesterolemia [61]. Serum levels of advanced glycation end products might be reduced through changes in diet [60] or in cooking practices. This reduction could explain the nonsignificant associations between the meat diet and the eGFR decline in this study, which was similar to those found in the Northern Manhattan Study [18].

In the subgroup analysis, significant associations for the vegetable pattern did not remain in participants without overweight or hypertension. This result might be due to the fact that the prevalence of cardiometabolic risk was high in this population; more than 50% of the participants had hypertension at baseline. Thus, those with hypertension, whether in treatment or not, were on a more cautious diet [62] and might have increased their consumption of vegetables [42]. High salt intake is an established risk factor for kidney function decline, mainly through its adverse effect on blood pressure and vascular health. Vegetables and fruit are high in potassium, and high potassium intake could also positively affect kidney function [14]. Hypertension might also be considered partially mediated by obesity [63] or sodium [41]; however, we did not have data on sodium or the sodium–potassium ratio for analysis. Additionally, although participants living in temporary houses or shelters had lower vegetable pattern scores, higher juice pattern scores, and the same meat pattern scores compared with other residents, we observed similar significant associations when stratified by post-disaster residence. 

One of the strengths of this study was that it had a large sample with repeated longitudinal measurements of kidney function, and the associations measured were more robust than in simple cross-sectional surveys. Further, we applied not only the eGFR cut-off level and proteinuria as the outcomes for measuring the associations but also yearly changes based on the trajectory analysis; both showed prominent significant results. 

This study had a few limitations. First, FHMS response rates remained at ≈27%; thus, the representativeness of the results might not be generalizable to the entire prefecture or the country’s population. Second, we could not compute the food amounts or nutrient amounts with energy adjustment, which would have helped us better elucidate the underlying mechanisms [63]. Meanwhile, a total of 19 food group items might not be able to determine correlations between specific foods [43]. Third, we computed the dietary pattern scores arising from FFQ surveys at baseline; thus, we could not clarify whether changes in the residence, employment, or dietary habits of participants during follow-up years impacted the associations measured [18,47]. Finally, the presence of residual confounding was possible, as in any observational study [27].

## 5. Conclusions

Our study suggests that the vegetable dietary pattern could be inversely associated with risk of impaired kidney function, including lower eGFR and proteinuria, whereas the juice dietary pattern could be positively associated with the risk of impaired kidney function. Continuous promotion of a balanced diet, particularly a vegetable diet rich in traditional Japanese foods, might be necessary to prevent the progression of impaired kidney function, thus reducing the burden of CKD with aging. Further validation studies or randomized controlled trials on these associations are needed, examining how individual foods influence kidney function. 

## Figures and Tables

**Table 1:** Factor loadings of dietary patterns identified by principal component method of factor analysis, FHMS, 2011 (*n* = 14,732).

Food Groups	Vegetable	Juice	Meat
White vegetables	**0.69**	0.14	0.22
Green vegetables	**0.65**	0.20	0.18
Tofu	**0.64**	0.13	0.06
Miso soup	**0.63**	−0.12	−0.09
Red/yellow vegetables	**0.63**	**0.30**	0.25
Fish	**0.51**	0.06	0.23
Fermented beans	**0.48**	0.14	−0.13
Fruit	**0.45**	0.41	0.01
Boiled beans	**0.38**	0.37	0.08
Rice	**0.34**	−0.22	−0.05
Vegetable juice	−0.02	**0.71**	0.004
Fruit juice	−0.01	**0.68**	0.08
Yogurt	0.22	**0.53**	0.004
Soybean milk	0.08	**0.40**	−0.04
Bread	−0.23	**0.35**	**0.31**
Milk	0.19	**0.34**	0.06
Beef/pork	0.15	−0.05	**0.74**
Ham/sausage	−0.01	0.07	**0.69**
Chicken	0.16	0.04	**0.68**
% Variance explained	3.26	2.21	1.81

FHMS, Fukushima Health Management Survey. Loadings with an absolute value more than 0.30 are shown in bold.

**Table 2:** Characteristics of participants at baseline in 2011, FHMS (*n* = 14,732).

	All	Men (*n* = 5964)	Women (*n* = 8768)	*p* Value
Age (years), mean (SD)	61.4 (10.0)	62.6 (9.9)	60.5 (9.9)	<0.001
Education ≥ vocational university, %	20.9	19.3	22.1	<0.001
Current smoker, %	13.3	24.9	5.4	<0.001
Current alcohol drinking, %	45.3	71.8	27.2	<0.001
Physical activity ≥ 2 times/week, %	42	45.1	39.9	<0.001
Distress scale ≥ 13, %	14.6	11.1	16.9	<0.001
Live at shelter/temporary house, %	39.8	39.1	40.3	0.094
BMI (kg/m^2^), mea(SD)	23.7 (3.4)	24.2 (3.1)	23.3 (3.5)	<0.001
BMI ≥ 25 kg/m^2^, %	32.2	38.2	28.1	<0.001
Hypertension, %	50.5	58.1	45.4	<0.001
SBP (mmHg), mean (SD)	131 (15.8)	133.5 (15.0)	129.3 (16.1)	<0.001
DBP (mmHg), mean (SD)	78.6 (10.1)	80.6.0 (9.9)	77.2 (10)	<0.001
Fast blood glucose (mg/dL), median (IQR)	97 (90, 105)	100 (93, 110)	95 (89, 102)	<0.001
Fast blood glucose ≥ 126 mg/dl, %	7	10	4.9	<0.001
HbA1C1 ≥ 6.5%, %	6.6	9.1	5	<0.001
LDL-C (mg/dL), mean (SD)	126.8 (31.7)	122.6 (31.9)	129.7 (31.3)	<0.001
LDL-C ≥ 140 mg/dL, %	33.2	29.1	36	<0.001
HDL-C (mg/dL), mean (SD)	60.8 (15.2)	56.1 (14.5)	64 (14.9.0)	<0.001
HDL-C < 40 mg/dL, %	5.7	9.8	2.8	<0.001
Triglycerides (mg/dL), median (IQR)	97 (69, 136)	106 (75, 152)	91 (66, 126)	<0.001
Triglycerides ≥ 150 mg/dL, %	19.5	25.9	15.1	<0.001
eGFR, mL/min/1.73 m^2^, median (IQR)	74 (67, 82)	73 (67, 82)	74 (68, 82)	<0.001
Vegetable pattern score, median (IQR)	0.01 (−0.68, 0.73)	−0.09 (−0.77, 0.65)	0.08 (−0.61, 0.78)	<0.001
Juice/milk pattern score, median (IQR)	−0.17 (−0.69, 0.47)	−0.33 (−0.84, 0.29)	−0.06 (−0.58, 0.58)	<0.001
Meat pattern score, median (IQR)	−0.21 (−0.67, 0.46)	−0.31 (−0.71, 0.34)	−0.14 (−0.63, 0.54)	<0.001

BMI, body mass index; DBP, diastolic blood pressure; eGFR, estimated glomerular filtration rate; FHMS, Fukushima Health Management Survey; HDL-C, high-density lipoprotein cholesterolemia; IQR, interquartile; LDL-C, low-density lipoprotein cholesterolemia; SBP, systolic blood pressure; SD, standard deviation.

**Table 3:** Kidney function in participants at follow-up years, 2012–2015, FHMS.

	2011		2012		2013		2014		2015		*p* Value
	(*n* = 14,732)		(*n* = 10,999)		(*n* = 9597)		(*n* = 8713)		(*n* = 8477)		
eGFR (mL/min/1.73 m^2^), mean (SD)	75.7	(11.0)	73.9	(11.9)	72.3	(11.5)	71.0	(11.5)	70.8	(11.7)	<0.001
eGFR (mL/min/1.73 m^2^) category, *n* (%)											<0.001
<60	0		889	(8.1)	1060	(11.1)	1195	(13.7)	1262	(14.9)	
60–90	13,131	(89.1)	9035	(82.1)	7777	(81.2)	6981	(80.1)	6673	(78.7)	
≥90	1601	(10.9)	1075	(9.8)	733	(7.7)	536	(6.1)	541	(6.4)	
Proteinuria											0.049
Negative	14,602	(99.4)	10,771	(97.9)	9358	(97.7)	8528	(97.8)	8254	(97.4)	
Trace	91	(0.6)	123	(1.1)	100	(1.0)	79	(0.9)	102	(1.2)	
Positive	0		95	(0.9)	104	(1.1)	97	(1.1)	115	(1.4)	
eGFR < 60 mL/min/1.73 m^2^ or proteinuria, *n* (%)	0		973	(8.8)	1143	(11.9)	1270	(14.6)	1350	(15.9)	<0.001
			**2011–2012**		**2012–2013**		**2013–2014**		**2014–2015**		***p* Value**
			**(*n* = 10,999)**		**(*n* = 7342)**		**(*n* = 6612)**		**(*n* = 6337)**		
Annual change of eGFR (mL/min/1.73 m^2^ per year), mean (SD)			−1.8	(7.3)	−1.2	(6.9)	−1.3	(6.2)	−0.3	(6.0)	<0.001
Annual change category, *n* (%)											<0.001
<−30%			6589	(59.9)	3954	(41.3)	3715	(42.6)	3034	(35.8)	
−30—< 15%			666	(6.1)	507	(5.3)	457	(5.2)	479	(5.6)	
≥15%			3744	(34.0)	2881	(30.1)	2440	(28.0)	2824	(33.3)	

eGFR, estimated glomerular filtration rate; FHMS, Fukushima Health Management Survey; SD, standard deviation.

**Table 4:** Cumulative incidence ratios (95% confidence intervals) of impaired kidney function among dietary patterns, 2012–2015, FHMS.

		eGFR < 60 (mL/min/1.73 m^2^)		Proteinuria		eGFR < 60 (mL/min/1.73 m^2^) or Proteinuria	
		CIR ^a^	95% CI	CIR ^a^	95% CI	CIR ^a^	95% CI
Vegetable							
Model 1	T1 (lowest)	1.00	Referent	1.00	Referent	1.00	Referent
	T2	0.97	0.88, 1.07	0.80	0.61, 1.04	0.95	0.87, 1.05
	T3	0.89	0.81, 0.98	0.67	0.51, 0.88	0.87	0.79, 0.95
	*P* for trend	0.013		0.005		0.001	
Model 2	T1 (lowest)	1.00	Referent	1.00	Referent	1.00	Referent
	T2	0.98	0.89, 1.08	0.80	0.62, 1.04	0.96	0.88, 1.06
	T3	0.90	0.82, 1.00	0.68	0.52, 0.90	0.88	0.80, 0.97
	*P* for trend	0.031		0.007		0.005	
Juice							
Model 1	T1 (lowest)	1.00	Referent	1.00	Referent	1.00	Referent
	T2	1.08	0.98, 1.19	0.97	0.74, 1.26	1.07	0.97, 1.17
	T3	1.20	1.09, 1.32	1.08	0.83, 1.41	1.19	1.09, 1.30
	*P* for trend	<0.001		0.543		<0.001	
Model 2	T1 (lowest)	1.00	Referent	1.00	Referent	1.00	Referent
	T2	1.07	0.97, 1.18	0.94	0.72, 1.22	1.05	0.96, 1.15
	T3	1.19	1.08, 1.31	1.04	0.79, 1.36	1.18	1.08, 1.29
	*P* for trend	<0.001		0.738		<0.001	
Meat							
Model 1	T1 (lowest)	1.00	Referent	1.00	Referent	1.00	Referent
	T2	0.97	0.89, 1.06	1.00	0.77, 1.31	0.97	0.89, 1.06
	T3	0.96	0.88, 1.06	1.17	0.90, 1.52	0.98	0.90, 1.08
	*P* for trend	0.459		0.214		0.809	
Model 2	T1 (lowest)	1.00	Referent	1.00	Referent	1.00	Referent
	T2	0.97	0.89, 1.07	1.02	0.78, 1.33	0.98	0.90, 1.06
	T3	0.98	0.89, 1.07	1.20	0.92, 1.55	1.00	0.92, 1.09
	*P* for trend	0.695		0.158		0.898	

CIR, cumulative incidence ratio; eGFR, estimated glomerular filtration rate; FHMS, Fukushima Health Management Survey; T, tertile. ^a^ Poisson regression, Model 1: adjusted for age (continuous), age^2^ (continuous) and sex; Model 2: model 1+ smoking history (never/former/current), alcohol drinking (never/occasional/regular), education (<occasional university/≥occasional university), physical activity (none/1 time per week/2–4 times per week/every day), distress scale (K6<13/≥13), residence (temporary house or shelter/others), overweight (no/yes), diabetes (no/yes), hypertension (no/yes), hyper-low-density lipoprotein cholesterolemia (no/yes), hypo-high-density lipoprotein cholesterolemia (no/yes), and hypertriglyceridemia (no/yes).

**Table 5:** Associations between dietary patterns and annual eGFR change, rising eGFR, and decreasing eGFR groups, 2012–2015, FHMS.

		Annual Change in eGFR (mL/min/1.73 m^2^ Per Year)		Increasing eGFR		Rapid Decline in eGFR	
		β ^a^	95% CI	OR ^b^	95% CI	OR ^b^	95% CI
Vegetable							
Model 1	T1 (lowest)	0	Referent	1.00	Referent	1.00	Referent
	T2	0.24	0.03, 0.44	0.96	0.86, 1.06	0.86	0.76, 0.98
	T3	0.27	0.06, 0.49	0.95	0.85, 1.06	0.83	0.73, 0.94
	*P* for trend	0.012		0.4		0.006	
Model 2	T1 (lowest)	0	Referent	1.00	Referent	1.00	Referent
	T2	0.23	0.02, 0.43	0.96	0.86, 1.06	0.88	0.77, 1.00
	T3	0.26	0.04, 0.47	0.94	0.84, 1.06	0.85	0.75, 0.98
	*P* for trend	0.019		0.422		0.009	
Juice							
Model 1	T1 (lowest)	0	Referent	1.00	Referent	1.00	Referent
	T2	−0.08	−0.28, 0.12	0.92	0.82, 1.02	1.11	0.98, 1.26
	T3	0.08	−0.12, 0.28	0.92	0.83, 1.03	0.99	0.87, 1.13
	*P* for trend	0.553		0.204		0.836	
Model 2	T1	1.00	Referent	1.00	Referent	1.00	Referent
	T2	−0.08	−0.28, 0.12	0.92	0.83, 1.03	1.10	0.98, 1.25
	T3	0.07	−0.13, 0.28	0.94	0.85, 1.05	1.00	0.88, 1.14
	*P* for trend	0.607		0.284		0.937	
Meat							
Model 1	T1 (lowest)	0	Referent	1.00	Referent	1.00	Referent
	T2	−0.06	−0.26, 0.14	0.93	0.83, 1.03	1.04	0.92, 1.18
	T3	0.04	−0.16, 0.24	1.07	0.96, 1.19	1.08	0.95, 1.23
	*P* for trend	0.62		0.099		0.256	
Model 2	T1 (lowest)	0	Referent	1.00	Referent	1.00	Referent
	T2	−0.07	−0.27, 0.13	0.92	0.83, 1.02	1.04	0.92, 1.18
	T3	0.03	−0.18, 0.23	1.06	0.96, 1.18	1.09	0.96, 1.23
	*P* for trend	0.732		0.095		0.176	

eGFR, estimated glomerular filtration rate; FHMS, Fukushima Health Management Survey; T, tertile. ^a^ Multiple linear regression, Model 1: adjusted for age (continuous) and sex; Model 2: model 1+ smoking history (never/former/current), alcohol drinking (never/occasional/regular), education (<occasional university/≥occasional university), physical activity (none/1 time per week/2–4 times per week/every day), distress (no/yes), residence (temporary house or shelter/others), overweight (no/yes), diabetes (no/yes), hypertension (no/yes), hyper-low-density lipoprotein cholesterolemia (no/yes), hyper-low-density lipoprotein cholesterolemia (no/yes), and hypertriglyceridemia (no/yes). ^b^ Polytomous logistic regression, covariates were the same as the analysis for the overall change of eGFR, with adding age^2^ and baseline eGFR level for an adjustment (continuous).

## Data Availability

The data presented in this study are available on request from the corresponding author. The data are not publicly available due to the privacy of participants from the radiation disaster areas.

## Supplementary Materials

The following are available online at https://www.mdpi.com/2072-6643/13/1/168/s1, Table S1: Characteristics of participants at baseline according to tertiles of dietary pattern scores, FHMS, 2011 (*n* = 14, 732). Table S2: Characteristics of participants in groups of eGFR progression, 2012–2015, FHMS. Table S3: Cumulative incidence ratios (95% confidence intervals) of impaired kidney function (eGFR < 60 mL/min/1.73 m^2^) among dietary patterns in participants without cardiometabolic risk at baseline, 2012–2015, FHMS. Table S4: Associations (coefficients) between dietary patterns and the annual change of eGFR (mL/min/1.73 m^2^) in participants without cardiometabolic risk at baseline, 2012–2015, FHMS. Table S5: Odds ratios (95% confidence intervals) of the eGFR rapid decline group among dietary patterns in participants without cardiometabolic risk at baseline, 2012–2015, FHMS.
