# Supplementary material for: Dietary Patterns and Progression of Impaired Kidney Function in Japanese Adults: A Longitudinal Analysis for the Fukushima Health Management Survey, 2011–2015
Source: Nutrients. 2021 Jan 7;13(1):168. doi: 10.3390/nu13010168 (PMC7827845; doi:10.3390/nu13010168)
Supplement: Supplementary file 1 [file nutrients-13-00168-s001.pdf]

nutrients MDPI

Supplementary materials

Table S1. Characteristics of participants at baseline according to tertiles of dietary pattern scores, 2011, FHMS (*n* = 14,732).

	All	Vegetable pattern			Juice pattern			Meat pattern		
		T1 (lowest)	T3 (highest)	*P* value	T1 (lowest)	T3 (highest)	*P* value	T1 (lowest)	T3 (highest)	*P* value
Age (years), mean (SD)	61.4 (10.0)	57.4 (9.7)	65.2 (8.9)	<0.001	60.1 (10.0)	62.5 (9.9)	<0.001	63.3 (9.0)	59.7 (10.7)	<0.001
Women, %	59.5	55.1	63.0	<0.001	50.6	66.8	<0.001	54.8	64.4	<0.001
Education ≥vocational university, %	20.9	21.9	20.0	0.071	17.5	24.4	<0.001	16.6	25.4	<0.001
Current smoker, %	13.3	19.7	7.5	<0.001	19.2	8.4	<0.001	11.5	13.6	<0.001
Current alcohol drinking, %	45.3	49.9	41.8	<0.001	53.9	38.7	<0.001	45.7	44.2	0.034
Physical activity ≥2 times/week, %	42.0	29.2	55.0	<0.001	33.4	50.2	<0.001	44.7	40.3	<0.001
Distress scale ≥13, %	14.6	15.7	14.2	0.018	13.7	15.6	0.026	14.7	15.3	0.073
Live at shelter/temporary house, %	39.8	42.1	37.1	<0.001	38.4	40.8	0.081	40.2	39.7	0.711
BMI (kg/m^2), mean (SD)	23.7 (3.4)	23.8 (3.5)	23.5 (3.3)	<0.001	23.8 (3.4)	23.6 (3.4)	0.004	23.8 (3.3)	23.5 (3.4)	<0.001
BMI ≥25 kg/m^2, %	32.2	33.9	30.3	0.002	32.9	30.5	0.021	34.2	30.4	<0.001
Hypertension, %	50.5	46.5	54.2	<0.001	50.8	50.1	0.744	55.6	46.1	<0.001
SBP (mm Hg), mean (SD)	131.0 (15.8)	129.8 (16.0)	132.0 (15.5)	<0.001	131.3 (16.1)	130.7 (15.6)	0.154	132.5 (15.6)	129.5 (16.1)	<0.001
DBP (mm Hg), mean (SD)	78.6 (10.1)	79.0 (10.3)	78.1 (9.8)	<0.001	79.2 (10.2)	78.0 (9.9)	<0.001	78.9 (9.9)	78.2 (10.2)	0.001

Fasting blood glucose (mg/dl), median (IQR)	97 (90, 105)	96 (90, 104)	97 (91, 106)	<0.001	97 (90, 105)	96 (90, 105)	0.628	98 (91, 107)	96 (90, 104)	<0.001
Fasting blood glucose ≥126 mg/dl, %	7.0	6.7	7.4	0.44	6.7	7.2	0.772	8.0	6.0	<0.001
HbA1C ≥6.5%, %	6.6	6.4	7.2	0.099	5.9	7.3	0.023	7.4	5.8	0.01
LDL-C (mg/dl), mean (SD)	126.8 (31.7)	128.4 (32.1.0)	125.4 (31.2)	<0.001	124.9 (31.9)	128.7 (31.6)	<0.001	126.0 (31.3)	126.9 (32.1)	0.08
LDL-C ≥140 mg/dl, %	33.2	35.5	31.2	<0.001	31.3	35.9	<0.001	31.9	33.7	0.063
HDL-C (mg/dl), mean (SD)	60.8 (15.2)	60.9 (15.5)	61 (15.0)	0.529	60.3 (15.3)	61.6 (15.1)	<0.001	59.4 (14.8)	62.5 (15.6)	<0.001
HDL-C <40 mg/dl, %	5.7	5.9	5.3	0.318	6.3	5.2	0.042	6.7	4.6	<0.001
Triglycerides (mg/dl), median (IQR)	97 (69, 136)	99 (70, 142)	95 (68, 130)	<0.001	98 (70, 140)	96 (69, 132)	0.044	101 (73, 141)	92 (66, 129)	<0.001
Triglycerides ≥150 mg/dl, %	19.5	21.9	17.0	<0.001	21.1	18.1	<0.001	21.3	17.1	<0.001
eGFR, mL/min/1.73 m^2, median (IQR)	74 (67, 82)	75 (68, 83)	73 (66, 81)	<0.001	75 (68, 83)	73 (66, 81)	<0.001	74 (67, 81)	75 (68, 83)	<0.001
Dietary pattern score, median (IQR)	-	−0.98 (−1.41, −0.68)	1.02 (0.73, 1.39)	-	−0.89 (−1.14, −0.69)	0.83 (0.48, −1.46)	-	−0.84 (−1.07, −0.67)	0.89 (0.46, 1.51)	-

BMI body mass index, DBP diastolic blood pressure, eGFR estimated glomerular filtration rate, FHMS Fukushima Health Management Survey, HDL-C high-density lipoprotein cholesterolemia, IQR interquartile, LDL-C low-density lipoprotein cholesterolemia, SBP systolic blood pressure, SD standard deviation, T tertile.

Table S2. Characteristics of participants in trajectory groups of eGFR progression, 2011–2015, FHMS (*n* = 14,732).

	Trajectory groups			
	**Increasing**	**Moderate decline**	**Rapid decline**	***P* value**
Age group, %				<0.001
40-49	21.6	62.6	15.9	
50-59	20.5	66.4	13.1	
60-69	17.4	69.8	12.8	
70-89	17.7	67.9	14.3	
Sex, %				<0.001
Men	21.1	67.1	11.7	
Women	17.3	67.8	15.0	
Education, %				0.015
<vocational university	18.5	67.8	13.7	
≥vocational university	67.8	18.5	13.7	
Smoking, %				<0.001
Never	17.7	68.4	13.9	
Former	21.0	67.2	11.8	
Current	21.0	63.5	15.6	
Alcohol drinking, %				<0.001
Never	17.2	68.4	14.4	
Occasional	19.0	67.0	13.9	
Regular	20.7	66.5	12.8	
Physical activity, %				0.033
0-1 time/week	19.4	66.6	14.1	
≥2 times/week,	18.2	68.6	13.2	
Distress scale, %				0.033
<13	19.1	67.4	13.5	
≥13	17.4	67.8	14.9	
Residence of post-disaster, %				<0.001
Own/relatives/apartment/rent	20.1	67.5	12.5	
Live at shelter/temporary house	17.1	67.6	15.4	
BMI (kg/m^2), %				0.803

<18.5	18.5	67.3	14.2	
18.5-24.9	18.6	67.7	13.7	
≥25	19.4	67.2	13.4	
Hypertension, %				0.001
No	18.9	68.5	12.6	
Yes	18.8	66.5	14.7	
Diabetes, %				<0.001
No	18.5	68.4	13.1	
Yes	18.9	63.2	17.9	
LDL-C ≥140 mg/dl, %				<0.001
No	19.0	66.4	14.6	
Yes	18.5	69.8	11.7	
HDL-C <40 mg/dl, %				0.679
No	18.9	67.4	13.7	
Yes	17.9	68.9	13.3	
Triglycerides ≥150 mg/dl, %				0.993
No	18.9	67.5	13.6	
Yes	18.9	67.4	13.7	
Vegetable dietary pattern score, %				<0.001
T1	20.0	65.7	14.3	
T2	18.6	67.9	13.5	
T3	17.9	68.9	13.1	
Juice dietary pattern score, %				0.027
T1	20.0	66.4	13.5	
T2	18.1	67.5	14.4	
T3	18.4	68.6	13.0	
Meat dietary pattern score, %				0.021
T1	18.8	68.3	13.0	
T2	17.8	68.3	13.9	
T3	20.0	65.9	14.1	

BMI body mass index, FHMS Fukushima Health Management Survey, HDL-C high-density lipoprotein cholesterolemia, LDL-C low-density lipoprotein cholesterolemia, T tertile.

Table S3. Cumulative incidence ratios (95% confidence intervals) of impaired kidney function (eGFR <60 mL/min/1.73 m^2) among dietary patterns in participants without cardiometabolic risk at baseline, 2012–2015, FHMS.

		Vegetable		Juice		Meat	
		**CIR[a]**	**95% CI**	**CIR[a]**	**95% CI**	**CIR[a]**	**95%CI**
BMI (18.5-24.0 kg/m^2)	T1 (lowest)	1.00	Referent	1.00	Referent	1.00	Referent
	T2	0.94	0.84, 1.06	1.01	0.89, 1.14	0.91	0.81, 1.02
	T3	0.89	0.79, 1.01	1.15	1.02, 1.30	0.91	0.81, 1.02
	P for trend	0.05		0.011		0.172	
No hyperten-sion	T1 (lowest)	1.00	Referent	1.00	Referent	1.00	Referent
	T2	0.99	0.85, 1.15	1.15	0.98, 1.34	0.96	0.83, 1.11
	T3	0.90	0.77, 1.05	1.25	1.08, 1.46	0.91	0.79, 1.06
	P for trend	0.136		0.005		0.245	
No diabetes	T1 (lowest)	1.00	Referent	1.00	Referent	1.00	Referent
	T2	0.98	0.89, 1.09	1.10	0.99, 1.22	0.94	0.85, 1.04
	T3	0.87	0.79, 0.97	1.19	1.07, 1.32	0.96	0.87, 1.07
	P for trend	0.009		0.002		0.546	
LDL-C <140mg/dL	T1 (lowest)	1.00	Referent	1.00	Referent	1.00	Referent
	T2	1.00	0.89, 1.11	1.01	0.90, 1.13	0.98	0.88, 1.09
	T3	0.88	0.78, 0.99	1.17	1.05, 1.30	1.02	0.92, 1.14
	P for trend	0.015		0.005		0.607	
HDL-C ≥40 mg/dL	T1 (lowest)	1.00	Referent	1.00	Referent	1.00	Referent
	T2	0.96	0.87, 1.05	1.05	0.95, 1.15	0.98	0.90, 1.08
	T3	0.88	0.80, 0.97	1.15	1.05, 1.27	1.00	0.91, 1.10
	P for trend	0.007		0.003		0.962	
Triglycerides <150 mg/dL	T1 (lowest)	1.00	Referent	1.00	Referent	1.00	Referent
	T2	1.00	0.90, 1.11	1.06	0.96, 1.18	0.98	0.89, 1.08
	T3	0.88	0.79, 0.98	1.19	1.07, 1.32	0.99	0.89, 1.09
	P for trend	0.008		0.001		0.804	

BMI body mass index, CIR cumulative incidence ratio, eGFR estimated glomerular filtration rate, FHMS Fukushima Health Management Survey, HDL-C high-density lipoprotein cholesterolemia, LDL-C low-density lipoprotein cholesterolemia, T tertile. [a] adjusted for age (continuous), age[2] (continuous), sex, smoking history (never/former/current), alcohol drink (never/occasional/regular), education (<occasional university/≥ occasional university), physical activity (none/1 time per week/2-4 times per week/every day), distress scale (K6<13/K6≥13), residence (temporary house or shelter/others), overweight (no/yes), diabetes (no/yes), hypertension (no/yes), hyper-LDL-C (no/yes), hypo-HDL-C (no/yes), and hypertriglycerides (no/yes), respectively.

Table S4. Associations (coefficients) between dietary patterns and the annual change of eGFR (mL/min/1.73 m² per year) in participants without cardiometabolic risk at baseline, 2012–2015, FHMS.

		Vegetable		Juice		Meat	
		**β [a]**	**95% CI**	**β [a]**	**95% CI**	**β [a]**	**95%CI**
BMI (18.5-24.0 kg/m²)	T1 lowest	1.00	Referent	1.00	Referent	1.00	Referent
	T2	0.23	−0.03, 0.50	−0.06	−0.32, 0.20	0.03	−0.23, 0.29
	T3	0.36	0.09, 0.63	0.35	0.09, 0.61	0.09	−0.17, 0.36
	P for trend	0.011		0.013		0.442	
No hypertension	T1 lowest	1.00	Referent	1.00	Referent	1.00	Referent
	T2	0.28	−0.01, 0.56	−0.01	−0.29, 0.27	−0.09	−0.39, 0.20
	T3	0.24	−0.07, 0.55	0.11	−0.19, 0.40	0.18	−0.12, 0.49
	P for trend	0.109		0.621		0.141	
No diabetes	T1 lowest	1.00	Referent	1.00	Referent	1.00	Referent
	T2	0.27	0.05, 0.49	−0.14	−0.36, 0.08	−0.04	−0.26, 0.17
	T3	0.34	0.12, 0.57	0.07	−0.15, 0.29	0.08	−0.14, 0.30
	P for trend	0.003		0.497		0.434	
LDL-C <140mg/dL	T1 lowest	1.00	Referent	1.00	Referent	1.00	Referent
	T2	0.30	0.04, 0.55	−0.10	−0.35, 0.15	−0.12	−0.37, 0.14
	T3	0.27	0.00, 0.54	0.08	−0.18, 0.35	0.01	−0.24, 0.27
	P for trend	0.044		0.646		0.86	
HDL-C ≥40 mg/dL	T1 lowest	1.00	Referent	1.00	Referent	1.00	Referent
	T2	0.22	0.01, 0.43	−0.08	−0.29, 0.13	−0.07	−0.28, 0.14
	T3	0.27	0.05, 0.50	0.08	−0.14, 0.29	0.03	−0.18, 0.24

	P for trend	0.016		0.601		0.748	
Triglycerides <150 mg/dL	T1 lowest	1.00	Referent	1.00	Referent	1.00	Referent
	T2	0.24	0.01, 0.47	−0.03	−0.26, 0.19	−0.09	−0.31, 0.14
	T3	0.19	−0.05, 0.43	0.15	−0.08, 0.38	−0.02	−0.24, 0.21
	P for trend	0.123		0.317		0.984	

BMI body mass index, eGFR estimated glomerular filtration rate, FHMS Fukushima Health Management Survey, HDL-C high-density lipoprotein cholesterolemia, LDL-C low-density lipoprotein cholesterolemia, T tertile. [a] Adjusted for age (continuous), age^2 (continuous), sex, smoking history (never/former/current), alcohol drink (never/occasional/regular), education (<occasional university/≥ occasional university), physical activity (none/1 time per week/2-4 times per week/every day), distress scale (K6<13/K6≥13), residence (temporary house/others), overweight (no/yes), diabetes (no/yes), hypertension (no/yes), hyper-LDL-C (no/yes), hypo-HDL-C (no/yes), and hyper-triglycerides (no/yes), respectively.

Table S5. Odds ratios (95% confidence intervals) of the eGFR rapid decline group among dietary patterns in participants without cardiometabolic risk at baseline, 2012–2015, FHMS.

		Vegetable		Juice		Meat	
		OR[a]	95% CI	OR[a]	95% CI	OR[a]	95% CI
BMI (18.5-24.0 kg/m^2)	T1 (lowest)	1.00	Referent	1.00	Referent	1.00	Referent
	T2	0.87	0.74, 1.02	1.16	0.99, 1.36	0.99	0.85, 1.17
	T3	0.79	0.67, 0.94	1.04	0.88, 1.23	1.07	0.91, 1.26
	P for trend	0.002		0.731		0.356	
No hypertension	T1 (lowest)	1.00	Referent	1.00	Referent	1.00	Referent
	T2	0.80	0.66, 0.97	1.13	0.94, 1.37	1.06	0.88, 1.29
	T3	0.98	0.80, 1.21	1.02	0.84, 1.24	1.07	0.88, 1.30
	P for trend	0.657		0.651		0.478	
No diabetes	T1 (lowest)	1.00	Referent	1.00	Referent	1.00	Referent
	T2	0.90	0.78, 1.04	1.06	0.92, 1.22	1.12	0.97, 1.29
	T3	0.82	0.70, 0.96	0.99	0.85, 1.15	1.11	0.96, 1.28
	P for trend	0.006		0.848		0.216	
LDL-C <140mg/dL	T1 (lowest)	1.00	Referent	1.00	Referent	1.00	Referent
	T2	0.85	0.73, 0.99	1.07	0.93, 1.24	1.05	0.90, 1.21
	T3	0.86	0.73, 1.01	1.00	0.86, 1.17	1.09	0.94, 1.27

	P for trend	0.03		0.817		0.222	
HDL-C ≥40 mg/dL	T1 (lowest)	1.00	Referent	1.00	Referent	1.00	Referent
	T2	0.87	0.77, 0.99	1.07	0.94, 1.22	1.04	0.91, 1.18
	T3	0.84	0.73, 0.96	0.98	0.86, 1.12	1.12	0.98, 1.27
	P for trend	0.006		0.854		0.082	
Triglycerides <150 mg/dL	T1 (lowest)	1.00	Referent	1.00	Referent	1.00	Referent
	T2	0.90	0.78, 1.04	1.06	0.92, 1.22	1.05	0.91, 1.21
	T3	0.80	0.68, 0.93	0.97	0.84, 1.12	1.14	0.99, 1.31
	P for trend	0.001		0.694		0.07	

BMI body mass index, eGFR estimated glomerular filtration rate, FHMS Fukushima Health Management Survey, HDL-C high-density lipoprotein cholesterolemia, LDL-C low-density lipoprotein cholesterolemia, T tertile. [a] Adjusted for age (continuous), age^2 (continuous), sex, smoking history (never/former/current), alcohol drink (never/occasional/regular), education (<occasional university/≥ occasional university), physical activity (none/1 time per week/2-4 times per week/every day), distress scale (K6<13/K6≥13), residence (temporary house or shelter/others), overweight (no/yes), diabetes (no/yes), hypertension (no/yes), hyper-LDL-C (no/yes), hypo-HDL-C (no/yes), and hyper-triglycerides (no/yes), respectively.
